# Supplementary material for: Are carbon emissions trading and green financial instruments synergistic? -Comprehensive quantitative research based on content analysis
Source: PLoS One. 2024 Mar 7;19(3):e0298601. doi: 10.1371/journal.pone.0298601 (PMC10919601; doi:10.1371/journal.pone.0298601)
Supplement: S1 Data — (ZIP) [file pone.0298601.s001.zip › Data/地级市Z_F工作报告.zip!/╜¡╦╒/│ú╓▌╩╨/└┤╫╘╣1⁄2╓┌║┼í╛┬φ┐╦╩2╛▌═°í┐_2015.docx]

各位代表：

现在，我代表常州市人民政府向大会作工作报告，请予审议，并请市政协各位委员和其他列席人员提出意见。

一、2014年工作回顾

过去的一年，面对错综复杂的经济形势和艰巨繁重的改革发展稳定任务，市政府在市委的正确领导和市人大、市政协的监督支持下，团结依靠全市人民，紧紧围绕主题主线，全力做好稳增长、调结构、促改革、重生态、惠民生各项工作，经济社会保持了持续稳定发展的良好势头，较好完成了年初确定的各项目标任务。

（一）经济发展稳中有进。预计全市实现地区生产总值4750亿元，比上年增长10%左右。完成公共财政预算收入433.88亿元，增长6.11%。实现固定资产投资3310亿元，增长16.1%，其中工业投入1680亿元，增长11%；服务业投入1622亿元，增长22.2%。预计完成规模以上工业增加值2440亿元，增长11%以上；实现社会消费品零售总额1800亿元，增长13%左右。完成外贸进出口总额287亿美元，其中出口214亿美元，增长5%。注册外资实际到账31亿美元。

（二）重大项目实现突破。积极开展“重大项目突破年”活动，共引进100亿元或10亿美元项目2个，50亿元或5亿美元项目8个。成功引进了东风、北汽、众泰等3个汽车整车项目，圆了常州多年未实现的汽车整车梦；总投资超过70亿元的博盈斯太尔发动机项目成功落户并实现首台样机下线；新誉集团与庞巴迪合作的飞机改装项目进展顺利，威翔航空通用飞机制造项目落户我市。项目推进机制进一步完善，每月召开重大项目推进会，及时督查项目建设情况。重点项目建设扎实推进，省、市重点项目累计完成投资707亿元，埃马克机床一期、诺贝丽斯汽车专用铝板材一期等一批重点项目竣工投产。

（三）转型创新取得实效。大力实施“三位一体”工业经济转型升级战略，十大产业链预计完成产值3500亿元，增长12%，占规模以上工业比重超过31%；130项“双百”重点项目完成投资210亿元，120家“双百”龙头骨干企业完成产值4700亿元，增长12%；构建了13家领军型创新企业、116家科技上市后备企业和986家高新技术企业组成的创新型企业集群。加快服务业提档升级，预计服务业增加值占地区生产总值的比重达46.5%左右。金融业规模持续扩大，全市银行业金融机构人民币存贷款余额分别增长6.5%、10.9%；金融商务区启动建设；企业股改上市稳步推进，实现IPO上市2家、新三板挂牌8家，全市上市企业总数达36家。旅游业发展势头良好，接待国内外游客4950万人次，实现旅游总收入648亿元，分别增长11.5%和14%。电子商务快速发展，积极创建国家电子商务示范城市，电子商务成交额达1150亿元，增长30%。科技创新步伐加快，科技企业孵化器、加速器面积达700万平方米，万人发明专利拥有量超12件，高新技术产业产值占规模以上工业产值比重达42.5%左右，“龙城英才计划”签约引进领军型创新创业人才155人，连续14年荣获“全国科技进步先进市”称号，成为国家知识产权示范城市，常州科教城蝉联中国最佳创业园区第二名并成为国家级两化深度融合试验区。文化产业做大做强，预计文化产业增加值占地区生产总值比重达5.5%左右。农业现代化加快推进，实现程度居全省第三，比上年前进一位。粮食继续丰产丰收，高效设施农业比重稳步提高。农业机械化水平达86%，位居全省前列。

（四）城乡环境不断优化。城乡基础设施加快建设，常溧高速公路、122省道、238省道常州段、金武路、延政路西延等工程加快实施，地铁1号线开工建设，青洋路高架北延、沪蓉高速青龙互通建成通车，桃园路、新堂北路、河海东路等城市道路改造工程竣工投用。生态绿城建设和国家森林城市创建工作取得实效，实施“增核”9600亩、“扩绿”17000亩、“联网”400公里，丁塘河湿地公园、凤凰公园、新龙生态林一期等公园绿地建成使用，老运河北侧慢行系统、高架沿线绿地基本建成。水利工程建设稳步实施，新沟河工程超额完成年度目标任务，中央财政小型农田水利重点县实现二轮全覆盖，国家级节水型城市创建顺利通过验收。信息基础设施日趋完善，无线综合覆盖率达98%，覆盖全市的4G通信网络已全面建成。全市所有乡镇建成电气化镇。生态文明建设扎实推进，2517个生态文明工程项目基本完成，节能降耗预警持续保持绿色，节能减排完成省下达目标任务。城市管理水平不断提高，“9313”综合整治工程进展顺利，市区完成综合整治项目348个，完成城中村整治114个，环城高架路沿线整治工作基本完成。城乡一体化步伐加快推进，全市土地承包经营权确权登记覆盖面达30%，“三大合作”改革不断深化，中心镇经济持续发展能力得到增强，美丽乡村示范村建设全面启动，首次对全市256个水稻种植面积超2000亩的行政村和16.4万亩市级生态公益林进行生态补偿，全市村级集体经济组织年收入同比增长10%，年收入千万元以上村达30个。

（五）改革开放迈出新步。产城融合改革试点工作进展顺利，规划和实施方案已经报省委、省政府审批。经济体制改革不断深化，重点推进简政放权、健全完善现代市场体系、国企国资改革、金融改革创新、深化财税体制、开放型经济体制等方面的改革，不断激发市场主体的活力和动力。推进工商登记和组织机构代码等制度改革，实行“先照后证、证照联办”，新批私营企业和个体工商户分别增长35.5%和15.1%。对外开放取得新成效，金坛经济开发区、溧阳经济开发区申报国家级经济开发区工作进展顺利，武进出口加工区、常州出口加工区升格为综合保税区加快推进。利用外资结构不断优化，制造业和生产性服务业实际到账外资占利用外资总额的73%，同比提升1个百分点。利用外资领域不断拓宽，新增融资租赁、商业保理等项目2个，新增2家外资企业总部和1家外资功能性机构。外贸综合服务平台正式建设并运行。积极推进通关作业无纸化改革，进出口通关效率分别提高77.6%、86.2%。常州机场实现一类口岸开放，成功开通香港、韩国首尔航线，机场旅客吞吐量达186万人次，增长21.6%。

（六）人民生活持续改善。居民收入稳定增长，预计城乡居民人均可支配收入分别增长9.3%和10.5%左右。就业创业取得实效，城镇新增就业13.5万人，扶持创业6500人，援助困难群体就业9000人。社会保障水平稳步提高，城乡大病医疗保险全面实施，连续第10年提高企业退休人员基本养老金，市区城乡低保标准和市区低保边缘困难家庭补助标准分别提高到620元/月和1500元/年，职工医保和城镇居民医保政策范围内住院医疗费用基金支付比例分别达80%和70%以上。养老事业加快发展，新增养老床位1671张，总数达3万张，市区护理型床位占养老床位总数的40%。住房保障和“三改”工作有序推进，新开工建设各类保障房22447套（户），发放住房公积金政策性低息贷款57亿元，中心城区中低收入住房困难家庭应保尽保；完成“三改”户数3152户，中心城区20个住宅小区低洼地改造工作全面完成。教育质量稳步提升，基础教育优质均衡发展，流动就业创业人员随迁子女公办吸纳比例达90%，中职学生对口单招本科达线率连续15年名列全省第一，高考本二以上录取率达70%，居全省前列。卫生事业加快发展，市一院综合病房大楼和市七院门急诊病房大楼投入试运行，市妇保院、市一院钟楼院区启动建设，各辖市区均创成省级慢性病综合防控示范区，城市居民在社区卫生服务机构门诊就诊比例达50%，农村居民县域内就诊率达85%。文化事业繁荣发展，中国大运河（常州段）成为世界文化遗产，国家历史文化名城申报迎检工作扎实推进，“文化100”、“四个演”、“社区天天乐”等文化活动全年超7000场次，观众超300万人次。体育事业蓬勃开展，我市在第18届省运会中获得的金牌、奖牌和总分均位居全省前列，7名运动员参加青奥会，共获得7枚金牌；“10分钟体育健身圈”建设高水平推进，戚墅堰区全民健身中心和新北区春江全民健身中心基本建成。加快基层社会治理创新，社区“减负增效”加快推进，全市城乡和谐社区达标率分别达98.7%和98.2%。着力办好民生实事，建设生态绿城、实施交通便民等十件为民办实事项目全面完成。深入推进精神文明和“平安常州”建设，法治建设满意度位居全省第一，各辖市区首次实现省级“双拥模范县（区）”满堂红，社会保持和谐稳定。与此同时，粮食供销、安全生产、国防动员、外事侨务、对台事务、审计统计、海关国检、质监药监、工商物价、司法行政、海事边防、邮政供电、档案方志、防震减灾、应急管理、民族宗教、妇女儿童、人口计生、残疾人事业、老龄事业和关心下一代等工作都取得了新成绩。

在推动经济社会发展的同时，我们不断加强自身建设，努力改进政府工作。扎实开展群众路线教育实践活动，“四风”问题得到有效解决。加快推进行政审批制度改革，形成“五张清单、四条主线、一个平台、六大改革”基本框架。精简下放行政审批事项，市级审批事项由2011年的380项减少到2014年的212项，精简比例达44.2%，提前完成本届政府任期内市政府部门审批项目减少三分之一以上的目标。清理并公布市政府各部门行政权力，精简比例达29.9%。全市机关事业单位在职人员首次实现负增长。开展建设项目审批流程再造，工业项目审批压缩时间三分之一以上，政府投资项目压缩时间近40%。惩治和预防腐败体系不断完善，政府系统廉政建设得到加强。着力推进依法行政，自觉接受市人大、政协及各民主党派的监督，办理人大代表建议、政协提案433件，妥善解决了一批经济社会发展方面的问题。

各位代表，去年各项成绩的取得，是全市上下在省委、省政府和市委的正确领导下，团结拼搏、攻坚克难、开拓创新的结果。在此，我代表市人民政府，向辛勤工作在各个领域的全市人民，向市人大代表、政协委员，向市各民主党派、工商联、各人民团体、各界人士和离退休老同志，向驻常人民解放军、武警官兵、公安政法干警和部省驻常单位，向所有关心、支持、参与常州建设和发展的中外投资者、科技工作者、务工人员和海内外朋友们，致以衷心的感谢和崇高的敬意!

在肯定成绩的同时，我们也清醒地认识到面临的挑战和工作中存在的问题，主要表现在：调结构、促转型面临较大压力，经济增长的质量和效益有待提高；部分企业特别是小微企业生产经营面临较大困难，公共财政预算收入和进口增长趋缓；大气环境质量有待进一步改善，节能减排任务依然繁重；有些政府部门和工作人员主动服务和担当意识不强，工作效率和服务质量需要进一步提高。对此，我们一定会高度重视，采取有针对性的措施，下大力气加以解决。

二、2015年工作安排

今年是全面深化改革的关键之年，是全面推进依法治市的开局之年，也是全面完成“十二五”规划的收官之年。今年政府工作的总体要求是：全面贯彻落实党的十八大、十八届三中、四中全会和习近平总书记系列重要讲话精神，按照市委十一届八次全会部署，紧紧抓住建设苏南国家自主创新示范区、产城融合综合改革试点的机遇，主动适应经济发展新常态，牢牢把握稳中求进工作总基调，深入开展“重大项目深化年”活动，加快转型升级，突出改革创新，推动产城融合，注重生态文明，加强民生保障，切实提高经济发展质量和效益，促进经济持续健康发展和社会和谐稳定。

今年经济社会发展的主要预期目标是：地区生产总值增长8.5%左右；公共财政预算收入增长6.5%左右；固定资产投资增长12%左右；社会消费品零售总额增长12%左右；外贸进出口总额和注册外资实际到账保持适度增长；城乡居民人均可支配收入增长9%以上；居民消费价格总水平涨幅控制在3%左右；城镇登记失业率控制在4%以内；全面完成省下达的节能减排目标任务。

为实现上述目标，我们将重点抓好八个方面工作：

（一）推进重大项目建设，增强经济发展后劲

1.加大重大项目招引力度。围绕十大产业链建设和现代服务业发展，紧盯世界500强企业、央企和大型民企开展招商，举办好“9.28”科技经贸洽谈会，引进一批产业层级高、投资规模大和带动能力强的大项目、好项目。重点在智能装备制造、碳材料、轨道交通、通用航空等产业链上引进一批关键性、龙头型、基地型的制造业大项目，提高产业竞争力。围绕提升现代服务业发展层次和区域影响力，瞄准国内外服务业龙头企业，着力招引一批健康医疗、电子商务、休闲旅游等服务业项目。

2.提高重大项目建设成效。全力加快重大项目建设进程，重点推动东风、众泰、北汽、斯太尔等重大项目投产达效，充分发挥重大项目对全市经济发展的支撑作用和对转型升级的带动作用。积极实施150个省、市重点项目，加快产业项目“六个一批”、社会事业和基础设施项目“四个一批”转化进程，着力提升投资完成率、新项目开工率、续建项目竣工率，年内完成投资634亿元。

3.完善重大项目推进机制。每月召开重大项目推进会，督查项目建设情况，及时协调解决项目推进中的难题，确保项目顺利实施。深入落实项目推进责任，强化地区和部门间的协调配合，加强与重大项目投资方的联系，推动项目早投产、早见效。进一步创新项目推进方式，千方百计破解项目推进中存在的用地、资金等方面的要素制约，为项目推进创造良好条件。

（二）加快经济转型升级，提高产业层次和水平

1.全力推进工业转型升级。深入实施“三位一体”工业经济转型升级战略，积极促进新兴产业裂变发展、传统优势产业提档升级、创新型企业加速成长。加快十大产业链升级步伐，按照“建链、补链、强链”的要求，大力发展整机整车等终端产品和共性技术、核心技术等关键环节，放大轨道交通、智能电网、太阳能光伏等产业链的竞争优势，推动新医药、新光源等产业链加快发展，促进碳材料和通用航空产业链快速崛起，培育壮大常州产业链的特色品牌，十大产业链产值占规模以上工业比重达32%以上。重点实施100个以上“双百”重点项目，鼓励和引导传统优势产业加大技改投入、推进“两化”深度融合，不断提高传统优势产业的技术水平、装备水平和产品附加值，增强传统产业市场竞争力。大力培育创新型企业集群，重点支持20家左右龙头企业成为创新型领军企业，科技型上市培育企业200家以上，高新技术企业1100家以上。

2.着力推进现代服务业发展。加快实施发展现代服务业三年行动计划，做强做大100家服务业优势企业，加快推进100个服务业重点项目和30个服务业集聚区建设，促进现代服务业快速发展。加快电子商务产业发展，扶持壮大本土电商企业，加快跨境电子商务服务体系建设，推动传统企业借助电子商务转型升级。促进健康服务业、养老服务业发展，加快建设常州生命健康产业园、海峡两岸健康产业合作示范区和西太湖医疗旅游先行区，努力把我市打造成长三角知名的健康之城。积极推动旅游业转型升级，推进国家智慧旅游公共服务平台建设，加快环球恐龙城航天乐园、东方盐湖城等项目建设进程。依托物联网、大数据、云计算等产业，提升服务业的信息化、智能化、现代化水平，提高服务业发展层次。年内服务业增加值占地区生产总值比重达48%左右。

3.扎实推进现代农业建设。紧紧围绕创建国家现代农业示范区和国家水产养殖综合标准化示范区，不断巩固农业综合生产能力，加快农业转型升级步伐，确保农业基本现代化进程位居全省第一方阵。结合全市城乡建设、土地利用和生态建设规划，加快编制农业“1185”工程布局规划，推动全市农业主导产业集聚集约发展。新建6万亩高标准农田，不断改善农田基础设施和耕地质量。开展稻麦亩产“吨粮市”创建活动，巩固提升粮食单产水平。推动现代农业产业园区建设，围绕农业种源、农产品加工、农产品物流等重点领域打造一批高水平的现代农业产业园区，新增10个市级以上现代农业产业园区。培育壮大以农产品加工为主的农业产业化龙头企业20家，形成一批富有核心竞争力的现代农业市场主体。加大农民教育培训力度，加快培育新型职业农民。

（三）加大科技创新力度，加快建设创新型城市

1.提升创新园区承载力。紧紧抓住苏南国家自主创新示范区建设的机遇，按照建设“创新驱动发展引领区、深化科技体制改革试验区、区域创新一体化先行区”的战略定位，加快创新园区建设步伐。围绕“一核两区三园多基地”的创新布局，支持科教城实施“333”工程，加快建设一流的高职教育园区和国际化产学研协同创新基地；常州高新区、武进高新区加快建设国内一流的国家级高新区；推动江苏中关村科技产业园、西太湖科技产业园、金坛华罗庚科技产业园加快进入省级高新区行列。加快建设“中以科技园”、“中德创新园区”等国际科技合作园区，提升园区国际化合作水平。

2.提高企业自主创新力。鼓励企业加大研发投入，建立研发机构，规模以上高新技术企业研发机构建设实现全覆盖，大中型工业企业研发机构建有率达95%以上，新增市级以上企业研发机构150家。积极推进创新成果专利化，着力形成一批自主创新核心技术，年内完成专利申请3万件，万人发明专利拥有量超12件。完善产学研合作常州模式，做优“5.18”展洽会和“天天5.18”品牌，新增产学研合作项目1000项以上，重点国际合作项目20项以上，促进创新要素向企业集聚。

3.增强创新创造协同力。支持行业骨干企业与大院大所、知名高校联合组建研发平台和科技创新战略联盟，开展协同创新，快速突破一批关键核心技术。鼓励在常高校围绕常州产业发展重点，与本土企业开展技术攻关合作。促进科技与金融有机结合，建立科技资源与金融资源有效对接机制，在加大财政资金投入的同时，充分利用资本市场，撬动社会资本增加科技创新投入。深入实施“龙城英才计划”和“杰出创新人才引育云计划”，引进培养一批掌握核心技术、能带动产业发展的领军人才。加大高技能人才培养引进力度，新增高技能人才1.8万人。完善人才激励机制，落实知识产权、科技成果转化为股权、期权等政策，吸引人才到我市创新创业。

（四）全面深化改革开放，增创体制机制优势

1.加快重点领域改革。进一步深化经济体制重要领域和关键环节改革，调动和激发市场主体的内生动力和活力。深入推进行政审批制度改革，重点围绕“服务企业、服务项目、规范市场、便民利民”四条主线，优化注册登记、项目审批、专业中介以及民生服务，加快构建高效规范、权责一致的为民服务体系和责任机制。全面深化国有企业和国有资产管理体制改革，调整优化国有经济布局结构，加快发展混合所有制经济，规范企业法人治理结构，提高国有资本运营效率。推进金融改革创新，加快金融商务区建设进程，增强江南银行、东海证券等机构综合实力，推动企业股改上市，力争5家企业上市，15家企业在新三板挂牌，大力发展金融租赁服务业，努力把我市建成区域性资本运作中心、资产管理中心和金融服务中心。推进传统媒体与新兴媒体融合发展，增强媒体引领力，深化国有文艺院团改革，提升院团精品生产能力和文化惠民服务水平。深化农村改革，全市基本完成农村土地承包经营权确权登记颁证的基础工作，确权登记覆盖面达70%左右，完成90%以上村的农村集体产权制度改革任务，开展农村集体建设用地和宅基地流转试点，推进农村产权交易市场建设，做好武进区“全国农村改革试验区”各项工作。

2.提升外资外贸发展水平。瞄准国际知名跨国公司和行业龙头企业，着力引进一批技术含量高、市场前景好、带动能力强的产业链项目，力争超10亿美元、5亿美元重大外资项目再有新突破，不断拓展利用外资新领域，吸引外商投资文化创意、旅游休闲、文化会展等产业。加大“走出去”力度，鼓励企业赴境外设立生产基地、开展项目投资和并购重组，加快德国埃森市“常州科技经济发展中心”建设步伐，支持有条件的企业走出去发展，提升国际化经营管理水平和综合竞争能力。加快“华贸通”等外贸综合服务品牌和出口基地建设，进一步发挥出口基地带动作用。推动区域通关一体化试点和电子口岸建设，加快推进关检合作“三个一、三个互”工作，提升贸易便利化水平，为外贸企业出口创造良好条件。

3.打造更高水平开放平台。积极参与和融入“一带一路”、长江经济带、沿海开发、苏南国家自主创新示范区等国家战略，推动各类开发区主动对接上海自贸区建设，积极做好产业配套、功能延伸等工作，构筑更高的对外开放新平台。积极推动国家级、省级开发区（高新区）结合自身实际，引进和建设十大产业链和现代服务业项目，把开发区建成全市重大项目建设的重要载体。推进开发区管理模式创新，完善开发区投融资机制，支持开发区通过社会资本特许经营、开发主体上市和发行债券等方式拓宽融资渠道。提升口岸开放水平，常州机场力争开通台湾地区、东南亚等航线，促进航空港、常州港、出口加工区等“区港联动”，加快建设水、陆、空、铁联动发展、客货运快速增长的立体口岸体系。加快推进城市国际化进程，提升城市国际文明礼仪水平等软环境，加强国际化人才引进和培养。

（五）着力推进产城融合，构建城乡发展新格局

1.推动产城融合发展。认真组织实施产城融合改革试点方案，加快探索实践，积极走出一条“以产兴城、以城促产、宜居宜业、融合发展”的改革发展常州之路。注重空间优化，加快构建产城融合“一纵三横”总体空间格局，重点推进城市中心区、常金统筹核心片区、西南门户片区、东部片区、沿江片区等“一中心四片区”产城融合发展。坚持统筹发展，把城市建设和产业发展有机结合起来，推进产业向高端化、集聚化、特色化迈进，促进城镇发展与人口集聚、功能完善相结合。积极推进产城融合示范试点建设，中心城区重点提升文化宫-南大街商贸集聚区功能，加快武进、新北城区商务商贸副中心建设，城市新片区重点推进西太湖科技产业园、新龙国际商务城和常州科教城省科技服务业示范区建设，推进东部地区发展提档升级，加快形成具有常州特色、可复制可推广的产城融合模式。推动体制机制创新，加快建立与产城融合发展相适应的产业升级、生态建设、考核评价等体制机制，为产城融合发展提供有力保障。

2.提高城乡建设管理水平。进一步推动综合交通运输体系建设，构筑常州交通区位优势，常溧高速公路、122省道常州东段、金武路、延政西路等实现通车，开工建设常宜高速公路、265省道常州段，争取沿江城际铁路、德胜河航道整治提升工程开工建设。加快完善城市路网，改善市民出行条件，精心组织实施轨道交通1号线，续建和新建劳动西路（长江路-龙江路）、光华路、福阳路（青洋路-北塘河西）等道路工程，加快金融商务区、皇粮浜公园周边道路建设步伐，对飞龙西路进行大修。实施23项旧城改造项目，改善提升城区形象。加快城市公共基础设施建设步伐，德胜河应急取水头部及泵站工程建成投用，戚墅堰污水处理厂三期厂区工程全面竣工，续建天然气利用三期工程。加快推进一流电网建设，完成12个乡镇重点示范区建设。推进重点水利工程建设，基本完成长江堤防防洪能力提升工程土建施工，全面完成千亩以上圩堤达标建设。深化城市长效管理，深入推进城市环境综合整治“9313”工程，对城郊结合部、城中村、老旧小区等区域实施综合整治，提高市区道路环卫机械化作业覆盖率，逐步将金坛、溧阳、武进、新北的镇区纳入长效管理考评范围，不断提升城市形象。

3.促进城乡一体化发展。加快推进新型城镇化和城乡发展一体化，组织编制辖市区“多规融合”空间规划和重点中心镇、重点村规划，优化城乡空间格局。强化国土资源管理，提高土地节约集约利用水平。加快推进薛埠、南渡、孟河、雪堰等重点中心镇建设步伐，完善一般镇的服务功能，提升全市新型城镇化建设水平。扎实推进富民强村，创新新型集体经济发展路径和实现形式，做大做强村级集体经济，力争全市村集体年收入同比增长10%以上，年收入千万元以上村集体累计达40个。稳步推进美丽乡村示范村建设，高起点规划、高标准建设一批具有江南农村特色的示范村，提高农村人居环境水平。开展新一轮茅山老区帮扶工程，增强茅山老区持续发展能力。

（六）加强生态文明建设，营造优美宜居环境

1.提升生态绿城建设水平。围绕创建国家森林城市和国家生态文明示范市，加快生态绿城建设步伐，逐渐形成植物多样、绿量充沛、连片成网、具有鲜明江南地域特色的生态绿化新格局。实施生态绿道建设工程，重点推进高架环线绿道和藻江河生态绿道等工程，对京杭运河两侧的人文旅游资源进行整合，建设水岸相融、资源相连的中心城区慢行系统。实施郊野公园建设工程，重点推进城北郊野公园、宋剑湖湿地郊野公园和郑陆郊野公园的扩容。实施城镇公园绿地建设工程，基本建成横塘河湿地公园，推进皇粮浜湿地公园、白荡河绿地建设。实施生态源保护建设工程，重点推进小黄山生态公益林品质提升等工程。实施生态廊道建设工程，重点推进新沟河延伸拓浚等工程。有序推进生态文明细胞工程建设，打造一批国家生态工业示范园区、生态乡镇等生态文明建设示范亮点。

2.提升大气环境质量。把改善提升大气环境质量作为一项重要的民生工程，确保PM2.5平均浓度较2013年下降7%。严格环境准入，不核准、备案“两高”项目和产能严重过剩行业的新增产能项目，加快落后产能淘汰，有序推进城市主城区重污染企业环保搬迁。优化能源结构，控制煤炭消费总量，提高耗煤项目准入门槛；全面整治燃煤锅炉，年内淘汰剩余50%燃煤小锅炉，完成禁燃区范围内所有燃煤设施淘汰或清洁能源替代。深化工业污染治理，钢铁、水泥行业分别从1月1日、7月1日起全面执行新排放标准，推动重点企业清洁生产改造，累计完成40%以上钢铁、水泥、化工、石化、有色金属等重点行业清洁生产审核。大力开展机动车尾气、扬尘、餐饮油烟、秸秆焚烧等污染整治，强化重污染天气应急管理，不断改善空气质量。

3.强化水土污染整治。深入推进太湖治水，严格控制入太湖河道水质，确保太湖水治理达到省考核要求。加强饮用水保障，建成投运长荡湖水厂20万吨/日一期工程，开工建设钱资荡生态清淤等工程，确保全市集中式饮用水源地水质100%达标。加强河道综合整治，对已整治的市河实施长效管理，提高河道水质。加强农业面源污染治理，积极开展畜禽养殖场综合整治。加强水环境基础设施建设，完成市江边污水厂中水回用和金坛市第二污水处理厂扩建等工程，开工建设邹区污水处理厂、溧阳市垃圾卫生填埋场一期封场等工程，推进市污水处理厂污泥焚烧项目，建成市餐厨废弃物综合处理一期工程。积极开展土壤污染防治和修复，全面完成耕地、集中式饮用水水源地土壤环境质量评估和污染源排查工作，对常隆及周边地块污染场地实施土壤修复。

（七）突出民生民本优先，推动社会事业协调发展

1.强化基本公共服务体系建设。完善就业创业服务体系，新增城镇就业8.5万人，开展城乡劳动者就业技能培训3万人，常州籍高校毕业生就业率达95%以上，扶持创业5500人。健全社会保障体系，推进“全民参保登记计划”，稳步提高社会保障水平，企业职工养老、城镇职工医疗保险分别新增参保5万人、2.5万人，城乡基本养老保险、医疗保险和失业保险综合覆盖率保持在98%以上，新农合人均筹资标准不低于580元，落实城乡低保标准自然增长机制。提升住房保障能力，新开工保障性安居工程15740套（户）、基本建成6350套（户），发放住房公积金政策性低息贷款45亿元，确保中心城区中低收入住房困难家庭公共租赁住房、经济适用住房应保尽保。完善养老服务体系，大力推进城乡社区居家养老服务中心（站）建设，年内城乡社区居家养老服务中心（站）覆盖率达100%，社会力量举办或经营的养老机构床位数占比超过50%，护理型床位占养老机构床位数超过40%，老年助餐点覆盖40%的城市社区。

2.提升发展各项事业。进一步推进教育现代化建设，加快教育资源布局调整和现代化学校建设，新开工省常中戚墅堰区分校、教科院附中等10所市属学校，续建五中、同济中学、丽华中学等9所市属学校，各辖市（区）实施学校基本建设项目35个，基本建成殷村职教园区，所有辖市（区）成为省义务教育优质均衡改革发展示范区。大力发展文化事业和文化产业，继续实施青果巷历史文化街区保护利用等申名重点工程，成功申报国家历史文化名城；提升文化惠民品质内涵，启动实施公共图书馆总分馆体系建设，打造“社区天天乐”、“文化100”、“江南心”等一批文化品牌，扎实推进省公共文化服务体系示范区创建；发展壮大文化产业，加大龙头文化企业和示范园区培育力度，全市文化产业增加值占地区生产总值比重达6%左右。推动医疗卫生事业发展，启动建设市三院公共卫生临床中心和市中医院急诊病房综合楼，加快市妇保院、市一院钟楼院区建设进程，以医疗联合体建设推进分级诊疗。创建国家级公共体育服务体系示范区，全面推进全市公共体育设施向社会免费开放或低收费开放，在全市设立100个科学健身指导示范点，提高全市公共体育服务水平。加快“智慧常州”建设，统筹推进三网融合发展，促进工业互联网、工业大数据等推广应用，新建住宅小区的光纤入户按照共建共享模式建设。加强食品药品监管，进一步提升食品药品安全保障水平。做好“十三五”发展规划编制工作，科学谋划常州未来发展。积极推进军民融合深度发展，广泛开展全民国防教育，不断加强民兵预备役建设，进一步提高双拥共建水平。支持工会、共青团、妇联等人民团体工作，进一步加强外事侨务、对台事务、档案方志、民族宗教、防灾减灾、应急管理、残疾人事业和关心下一代等工作。

3.加强社会治理方式创新。加强精神文明建设，充分发挥常州道德讲堂促进社会和谐的载体作用，巩固文明城市建设成果，弘扬向上向善的社会风尚。严格执行社区事务准入制度，巩固提升社区“减负增效”成果，“政社互动”覆盖全市所有街道（镇），不断提升社区自治水平。建立健全社会矛盾预警机制、利益表达机制、协商沟通机制、救济救助机制，夯实信访工作基层基础，依法维护群众权益，解决好群众最关心、最直接、最现实的利益问题。深入开展“民生警务”，推进公安基础信息化、警务实战化、执法规范化、队伍正规化“四项建设”，加快实施新一轮社会治安防控体系建设，不断提升“平安常州”建设水平。强化质量监管，创建“全国质量强市示范城市”。切实加强安全生产工作，建立健全“党政同责、一岗双责”的安全生产责任体系，落实企业安全生产主体责任，坚决遏制重特大安全事故发生。

与此同时，今年我们将办好以“三优三安两提升”（优质教育、优质医疗、优质养老、安心食品药品、安心饮用水、安心社会治安、提升生态绿城建设水平、提升大气环境质量）为重点的十件民生实事，让广大市民更多更好地享受常州改革发展的成果。

（八）大力建设服务型政府，全面提升行政服务效能

1.加快转变政府职能。加大简政放权力度，进一步精简审批事项、压缩审批时间、提升行政服务效能。按照“机构精简、职能明晰、资源集约”的原则，全面完成市县政府机构改革，积极推进“一办四中心”建设，加快构建覆盖全市、上下联动、功能完善、运行高效的四级政务服务体系。有序推进政府职能转移，将部分行业管理服务类、社会事务类和专业技术管理服务类事项转移给社会组织承担。完善行政权力网上公开透明运行机制，深入推进政府信息公开，实现财政预决算、部门预决算和“三公”经费除涉密部门和涉密事项外全部公开。

2.大力推进依法行政。进一步规范行政行为，完善政府权力清单制度和责任清单制度，加快建设职能科学、权责法定、执法严明、公开公正、廉洁高效、守法诚信的法治政府。健全依法决策机制，认真落实公众参与、专家论证、风险评估、合法性审查、集体讨论决定重大行政决策法定程序，建立重大决策终身责任追究制度及责任倒查机制。深化行政执法体制改革，推进严格规范公正文明执法。自觉接受人大的法律监督、工作监督和政协的民主监督，加强审计监督，重视舆论监督、公众监督。

3.切实改进工作作风。巩固提升群众路线教育实践活动成果，健全完善深入基层调查研究、服务基层群众等制度，进一步改进文风会风，促进作风建设制度化、规范化、常态化。大力弘扬奋发进取、敢于担当、勇争一流的创新创业精神，雷厉风行开展工作，提高工作执行力和落实力。切实贯彻党风廉政建设责任制，强化权力运行制约和监督，坚决查办各类违法违纪案件，努力实现干部清正、政府清廉、政治清明。

各位代表，做好今年的工作任务艰巨、责任重大。让我们紧密团结在以习近平同志为总书记的党中央周围，在省委、省政府和市委的坚强领导下，紧紧依靠全市人民，抢抓机遇，锐意进取，真抓实干，为推动常州经济社会发展迈上新台阶而努力奋斗！
